# Preoperative vitamin D level as a post-total thyroidectomy hypocalcemia predictor: a prospective study

**DOI:** 10.1016/j.bjorl.2019.07.001

**Published:** 2019-08-06

**Authors:** Carlos Segundo Paiva Soares, José Vicente Tagliarini, Gláucia M.F.S. Mazeto

**Affiliations:** a Universidade Estadual Paulista (Unesp), Faculdade de Medicina de Botucatu, Departamento de Oftalmologia, Otorrinolaringologia e Cirurgia de Cabeça e Pescoço, Botucatu, SP, Brazil; b Universidade Estadual Paulista (Unesp), Faculdade de Medicina de Botucatu, Departamento de Medicina Interna, Botucatu, SP, Brazil

**Keywords:** Parathyroid hormone, Thyroidectomy, Vitamin D

## Abstract

**Introduction:**

Hypocalcemia is one of the most common complications after total thyroidectomy. Preoperative serum vitamin D concentration has been postulated as a risk factor for this complication. However, the subject is still controversial and the role of vitamin D in the occurrence of hypocalcemia remains uncertain.

**Objective:**

To evaluate the capability of preoperative vitamin D concentrations in predicting post-total thyroidectomy hypocalcemia.

**Methods:**

Forty-seven total thyroidectomy patients were prospectively evaluated for serum 25(OH) vitamin D, calcium and parathyroid hormone before surgery, Calcium every 6 hours, and parathyroid hormone 8 hours post-operatively. Patients were divided according to postoperative corrected calcium into groups without (corrected calcium ≥8.5 mg/dL) and with hypocalcemia (corrected calcium <8.5 mg/dL), who were then evaluated for preoperative 25(OH) vitamin D values.

**Results:**

A total of 72.3% of cases presented altered 25(OH) vitamin D preoperative serum concentrations and 51% evolved with postoperative hypocalcemia. The with and without hypocalcemia groups did not differ for preoperative 25(OH) vitamin D (*p* = 0.62). Univariate analysis showed that age (*p* = 0.03), postoperative PTH concentration (*p* = 0.02), and anatomopathological diagnosis of malignancy (*p* = 0.002) were predictors of postoperative hypocalcemia. In multivariate analysis only parathyroid hormone in postoperative (*p* = 0.02) was associated with post-total thyroidectomy hypocalcemia.

**Conclusion:**

Preoperative serum concentrations of 25(OH) vitamin D were not predictors for post-total thyroidectomy hypocalcemia, whereas postoperative parathyroid hormone influenced the occurrence of this complication.

## Introduction

Total thyroidectomy is one of the most common operations performed on the head and neck worldwide. Hypocalcemia is one of its most frequent complications which can lead to serious repercussions and even death.^1^ Its exact prevalence is difficult to establish, bearing in mind the wide range of definitions and the differences in sampling.^2^

Despite the most frequent etiology of post-total thyroidectomy hypocalcemia being hypoparathyroidism, secondary to intraoperative direct removal or devascularization of the parathyroid,^3^ other factors seem to predispose to this clinical-laboratory condition, such as hemodilution, calcitonin liberation, advanced age, Graves’ disease, surgical technique, and surgeon experience.^4^, ^5^ Recently some authors have suggested that serum vitamin D concentrations could present a risk factor for hypocalcemia development.^2^, ^4^, ^6^, ^7^, ^8^ Yamashita et al. reported that Graves’ disease patients presenting vitamin D deficiency and elevated alkaline phosphatase levels were at higher risk of developing post-thyroidectomy tetany.^9^ Al-Khatib et al. observed that patients with preoperative 25(OH) Vitamin D (25OHD) levels lower than 25 nmoL/L had a 7.3 times higher risk of developing post-thyroidectomy hypocalcemia.^2^ However, the role of this level as a predictor for hypocalcemia is still controversial. Falcone et al. retrospectively evaluating 264 patients, concluded that 25OHD did not predict a drop in post-operative calcium.^3^ Lang et al. reported that preoperative 25OHD deficit (values below 20 ng/mL) did not increase the post-thyroidectomy hypocalcemia rate.^10^ Some of the reasons for these discordant results could be the criteria used to diagnose hypocalcemia, cut points for 25OHD, and study group heterogeneity.

In light of this, the main objective of our study was to evaluate the potential for preoperative serum vitamin D concentrations in predicting postoperative hypocalcemia in a homogenous group of patients submitted to total thyroidectomy.

## Methods

In this prospective observational study, between May 2017 and November 2018 we evaluated 47 patients submitted to total thyroidectomy in a tertiary hospital who met the selection criteria described below. Approval was obtained from the local Research Ethics Committee (Approval: 2,046,729) and all patients were informed and made clear about the study. They all signed the Informed Consent Form.

Each surgery was performed by the same team (C.S.P.S. & J.V.T.) and the standard surgical technique involved capsular dissection of the thyroid gland with careful identification and conservation of the parathyroid glands, together with the recurrent laryngeal nerve.

Inclusion criteria were patients aged between 18 and 85 years undergoing total thyroidectomy due to malignant or benign thyroid disease. Exclusion criteria were cases with uncontrolled hyper- or hypothyroidism, parathyroid disease, those who had undergone any type of dialytic procedure and those who were on medication which altered serum calcium concentrations.

The following general data were collected for sample characterization: gender, age (in years), thyroid volume by ultrasonography exam (in cm^3^), anatomopathological diagnosis (whether malignant or not), and whether central neck lymphadenectomy was performed during surgery or not.

The main outcome evaluated was the occurrence of hypocalcemia in the first 48 h after surgery, while the main variable of interest was preoperative serum concentration of 25OHD. All patients also had blood samples taken at admission for Thyroid Stimulating Hormone (TSH), free thyroxin (T4L), serum calcium, and protein fractions and Parathyroid Hormone (PTH). After surgery, patients were retested for levels of PTH 8 h post-total thyroidectomy and corrected calcium every 6 h post-operatively for 48 h. The Payne formula was used to adjust calcium values, where corrected calcium = calcium + 0.8 (4-albumin).

Patients were classified according to preoperative serum Vitamin D concentration, where values of 25OHD ≥ 30 ng/mL were considered normal and <30 ng/mL as altered.^11^ Patients were also subdivided into groups with and without hypocalcemia 48 h after surgery where hypocalcemia was considered when corrected calcium <8.5 mg/dL in the first 48 h postoperative. Groups with and without hypocalcemia and with normal or altered 25OHD were compared considering the above listed parameters.

### Statistical analysis

Descriptive analysis of data was performed with frequency and percentages for qualitative variables and means and standard deviations for quantitative variables. Comparison of variables with symmetrical distribution was made using the Student’s *t-*Test. Variables with asymmetric distribution were adjusted using a generalized linear model with gamma distribution followed by multiple comparisons. The same was performed for vitamin D. To verify the association between explanatory variables and hypocalcemia, we applied the Chi squared or Fisher’s exact test when necessary. Finally, to verify the factors which influenced outcome, we applied an adjusted univariate logistic regression model and variables presenting *p* < 0.15 were used in a multivariate logistic regression model. The level of significance adopted was 5%. For all analyses we used SAS version 9.4.

## Results

From the patients evaluated, 39 (83%) were female. Mean (±Standard Deviation) age of the sample was 50 (±14.5) years. Mean thyroid volume was 34.2 (±46.5) cm^3^, with malignancy by anatomopathological exam being found in 28 (60.9%) patients, of which 7 (25%; 14.9% of the total) were submitted to central neck lymphadenectomy.

Twenty-four (51%) patients developed hypocalcemia in postoperative (Table 1). The hypocalcemia patients presented lower age (*p* = 0.03) and thyroid volume (*p* = 0.03) than the normocalcemic group. Anatomopathological malignancy diagnosis was more frequent in the hypocalcemia group (*p* = 0.001). PTH concentrations in postoperative were lower in the hypocalcemic than normocalcemic group (*p* = 0.004). No statistical difference was seen between groups for gender, presence or absence of central neck lymphadenectomy, corrected calcium, PTH, or preoperative 25OHD.

**Table 1:** Comparison between the groups with and without postoperative hypocalcemia.

Variables	Normocalcemia	Hypocalcemia	*p*-value
	n = 23	n = 24	
Female (%)	82.6	83.3	0.94
Age (years)^a^	54 (±14.3)	45 (±13.5)	0.03
Thyroid volume (cm^3^)^a^	46.6 (±62)	23.6 (±24.1)	0.03
CL (%)	4.3	25	0.09
Diagnosis of cancer (%)	36.6	83.3	0.001
PTH PreO (pg/mL)^a^	32.5 (±16.7)	37.8 (±19.8)	0.32
CaC PreO (mg/dL)^a^	9.69 (±0.43)	9.54 (±0.46)	0.23
25OHD PreO (ng/mL)^a^	26.5 (±8.1)	25.5 (±5.9)	0.62
PTH PO (pg/mL)^a^	24.8 (±23.3)	9.7 (±8.7)	0.004

Statistical tests: Student’s *t* Test and chi-square; level of significance: *p* < 0.05.

CaC, Corrected Calcium; CL, Central Neck Lymphadenectomy; n, Number; PO, Postoperative; PreO, Preoperative; PTH, Parathyroid Hormone; 25OHD, Vitamin D.

a Values expressed as mean (± Standard Deviation).

Univariate analysis revealed that predictors of hypocalcemia in the postoperative period were age, PTH concentration postoperatively, and anatomopathological diagnosis of malignancy (Table 2). Serum 25OHD concentrations were not predictors of this outcome. Considering age, performance of central neck lymphadenectomy, diagnosis of malignancy and PTH postoperatively, multivariate analysis only showed an association between the latter and post-total thyroidectomy hypocalcemia (Table 3).

**Table 2:** Univariate logistic regression in the prediction of hypocalcemia after total thyroidectomy.

Variables	OR	CI	*p*-value
Gender	1.053	0.230–4.82	0.94
Age (years)	0.953	0.911–0.997	0.037
Thyroid volume (cm^3^)	0.986	0.968–1.006	0.16
CL (%)	7.329	0.807–66.569	0.07
Diagnosis of cancer	8.750	2.199–34.813	0.002
PTH PreO (pg/mL)	1.017	0.984–1.050	0.31
CaC PreO (mg/dL)	0.442	0.115–1.709	0.23
25OHD PreO (ng/mL)	0.979	0.901–1.063	0.61
PTH PO (pg/mL)	0.922	0.859–0.990	0.02

OR, Odds Ratio; CI, Confidence Interval, level of significance: *p* <  0.05; CaC, Corrected Calcium; CL, Central Neck Lymphadenectomy; PO, Postoperative; PreO, Preoperative; PTH, Parathyroid hormone; 25OHD, Vitamin D.

**Table 3:** Multivariate logistic regression in the prediction of hypocalcemia after total thyroidectomy.

Variables	OR	CI	*p*-value
Age (years)	0.986	0.924–1.051	0.6625
CL (%)	5.217	0.034–804.815	0.5205
Diagnosis of cancer	3.650	0.214–62.134	0.3707
PTH PO (pg/mL)	0.901	0.813–0.997	0.0440

OR, Odds Ratio; CI, Confidence Interval, level of significance: *p* < 0.05; CL, Central Neck Lymphadenectomy; PO, Postoperative; PTH, Parathyroid Hormone.

Thirty-four patients (72.3%) presented altered preoperative serum 25OHD levels: 7 (14.9%) <20 ng/mL and 27 (57.4%) ≥20 and <29 ng/mL. Thirteen patients (27.7%) showed levels ≥30 ng/mL. Mean (±SD) serum 25OHD levels for altered and normal vitamin D groups were 22.79 (±4.95) and 34.53 (±3.73) ng/mL respectively. Groups with altered and normal 25OHD levels displayed different preoperative PTH levels (*p* = 0.01), and no significant differences for preoperative corrected calcium (*p* =  0.55), PTH in postoperative (*p* = 0.74) and frequency of patients evolving to hypocalcemia in postoperative (*p* = 0.11) (Table 4).

**Table 4:** Comparison between groups with altered and normal concentration of 25OHD.

Variables	Altered^a^	Normal^b^	*p*-value
	n = 34	n = 13	
Hypocalcemia (%)	58.8	30.7	0.11
PTH PreO (pg/mL)^c^	39.21 (±19.03)	24.9 (±11.6)	0.01
CaC PreO (mg/dL)^c^	9.64 (±0.46)	9.55 (±0.42)	0.55
PTH PO (pg/mL)^c^	17.43 (±22.05)	19.34 (±13.57)	0.74

Statistical tests: Student’s *t* Test and Chi-Square; level of significance: *p* < 0.05.

CaC, Corrected Calcium; n, Number; PO, Postoperative; PreO, Preoperative; PTH, Parathyroid Hormone.

a Patients with 25OHD ≥ 30 ng/mL.

b Patients with 25OHD < 30 ng/mL.

c Values expressed as mean (± Standard Deviation).

## Discussion

In this study, evaluating a group of relatively homogenous patients submitted to total thyroidectomy, we observed that preoperative serum 25OHD concentrations were not predictors of post-surgery hypocalcemia. The calcium metabolism depends, among other things, on PTH and Vitamin D action. On the one hand, the hormone promotes renal calcium reabsorption, while on the other hand the vitamin stimulates intestinal calcium absorption.^6^ Both act in an extremely integrated manner with their ultimate main objective: to maintain normocalcemia. Thus, Vitamin D deficiency with a consequent reduction in intestinal calcium absorption leads to the compensatory secondary hyperparathyroidism with hypertrophy of the parathyroid glands and increased bone and renal calcium reabsorption induced by PTH. In our study this compensatory PTH response was confirmed by the higher preoperative PTH levels in the group with 25OHD < 30 ng/dL. Interestingly, this occurred even with mean levels of 25OHD compatible with insufficiency and not with Vitamin D deficiency in the altered group.^11^ It is important to emphasize that the majority of patients evaluated were found with levels below 30 ng/mL, and of these most were between 20 and 29 ng/mL. Although, more recently the cut-off level for 25OHD normality has been questioned with a tendency to consider a value of 20 ng/mL, our study showed that PTH levels were much higher even when vitamin D levels were not so low.^12^

Despite the above, in this study we did not observe a relationship between post-total thyroidectomy hypocalcemia and preoperative 25OHD levels. There was no difference in serum 25OHD levels between patients with normocalcemia and those with hypocalcemia, and there was no difference in hypocalcemia evolution percentage between those with normal and low serum 25OHD concentrations. Theoretically, patients with reduced serum Vitamin D levels are more prone to develop hypocalcemia due to a higher dependency on PTH induced bone and renal reabsorption mechanisms.^3^ Various studies have attempted to correlate Vitamin D deficiency with post-total thyroidectomy hypocalcemia, however this theme is still controversial. Griffin et al., retrospectively analyzing 121 patients submitted to total thyroidectomy, did not find a relationship between post-total thyroidectomy hypocalcemia, thyroidectomy totalization, and Vitamin D deficiency.^13^ Danan and Sohnka, after evaluating 67 patients, concluded that those who had 3 or more parathyroid glands identified intraoperatively, associated with serum concentrations of 25OHD lower than 25 ng/mL, presented a 5.8 times higher risk of developing hypocalcemia after surgery.^6^ The reasons for these conflicting results could be due to differences in the biochemical cut-off levels used, as well as in the patient's individual characteristics. It is known, for example that the risk of hypocalcemia would be higher in patients with a tendency toward postoperative hungry bone syndrome (e.g., thyroidectomy due to severe disease or associated to hyperparathyroidism) and with an important vitamin D deficit.^9^ In our study patients were rigorously selected for both morbidities or medication which could influence the calcium metabolism, and as already discussed, did not present severe Vitamin D deficiency.

Although other parameters frequently associated with hypocalcemia have initially shown a relationship to this outcome, only postoperative PTH remained significant in multivariate analysis. Therefore, the hormone collected 8 h after total thyroidectomy was the only predictive factor for postoperative hypocalcemia. This finding agrees with those reported by other authors,^14^, ^15^ including a recent metanalysis which observed that transitory hypocalcemia was related to low PTH between 30 min and 5 days after surgery.^16^

The main limitation of our study is the relatively modest sample size which precluded the analysis of a narrower interval of 25OHD levels. However, our study has the merit of the prospective analysis, besides the strict criteria used to exclude outcome confounding factors.

## Conclusion

We concluded that serum 25OHD levels were not predictors of postoperative hypocalcemia in the group of studied patients. The influence of postoperative PTH seen on hypocalcemia occurrence stimulates the need for more studies focused on levels of this hormone with a view to better understand the hormonal dynamics and the processes involved in reducing post-total thyroidectomy hypocalcemia.

## Conflicts of interest

The authors declare no conflicts of interest.
